# Detection of African Swine Fever Virus Genotype II in West Africa (2020) and Its Co-Circulation With Endemic Genotype I: Implications for Pig Production

**DOI:** 10.1155/tbed/5396227

**Published:** 2025-06-10

**Authors:** Irene Kasindi Meki, Adeyinka Jeremy Adedeji, Lalidia Bruno Ouoba, Yao Mathurin Koffi, Adama Diakité, Tirumala B. K. Settypalli, Lamouni Habibata-Zerbo, Kouamé Valère Kouakou, Mohamed Adama Diakité, Charles Masembe, Moctar Sidi, Thierry Ouattara Douyeri, Fatoumata Dembelé, Helen E. Luka, Sandaogo Hamidou-Ouandaogo, Christiane Dembelé, Rebecca Weka, Gregorie Bazimo, Martin Dakouo, Toyin A. Olubade, Mariétou Guitti-Kindo, Chaka Traoré, Olushola Gamra, Dominique Guigma, Cheick Abou Kounta Sidibé, Dupe A. Hambolu, Drabo Dji-tombo Adama, Boubacar Madio dit Aladiogo Maïga, Mary A. Ogunleye, Ayokanmi Toluhi, Nanven Maurice, Emmanuel Couacy-Hymann, Pam D. Luka, Giovanni Cattoli, Charles E. Lamien

**Affiliations:** ^1^Animal Production and Health Laboratory, Joint FAO/IAEA Centre of Nuclear Techniques in Food and Agriculture, Department of Nuclear Sciences and Applications, International Atomic Energy Agency, Wagramer Strasse 5, P.O. Box 100, A-1400, Vienna, Austria; ^2^National Veterinary Research Institute, PMB 01, Vom, Nigeria; ^3^College of Natural Sciences, Makerere University, Kampala, Uganda; ^4^Laboratoire National d'Elevage (LNE), Ouagadougou, Burkina Faso; ^5^Department of Production and Animal Health, Centre Universitaire Polytechnique de Dori, Université Thomas SANKARA, Ouagadougou, Burkina Faso; ^6^CNRA/LIRED, BP 446 Bingerville, Côte d'Ivoire; ^7^Laboratoire Central Vétérinaire, Bamako, Mali; ^8^Federal Ministry of Livestock Development, Nigeria; ^9^Ministry of Agriculture, Ikeja, Lagos State, Nigeria; ^10^Istituto Zooprofilattico Sperimentale delle Venezie, Legnaro, Italy

**Keywords:** CVR variants, food security, p72, phylogenetic analysis, serogroup, surveillance

## Abstract

African swine fever (ASF) is a highly devastating disease of domestic pigs caused by the ASF virus (ASFV). Historically, only ASFV Genotype I was known to circulate in West Africa. However, Genotype II has recently emerged in Nigeria, Ghana, and Benin for the first time. Between 2017 and 2023, suspected ASF outbreaks were reported in Burkina Faso, Côte d'Ivoire, Nigeria, and Mali. The source, extent, and spread of these ASF outbreaks remain unknown. Samples collected from 2017 to 2023 were analyzed using real-time qPCR and characterized using five ASFV gene segments: partial gene of the B464L (p72), full length E183L (p54), central variable region (CVR) within B602L, EP402R (CD2v), and intergenic region (IGR) between I73R and I329L genes. ASF was confirmed in 12 Nigerian states and in seven, eight, and two provinces of Burkina Faso, Côte d'Ivoire, and Mali, respectively. Phylogenetic analysis of B646L (p72), E183 (p54), and CD2v genes of ASFV revealed that Genotype I, Serogroup 4, caused the initial outbreaks in these countries, followed by Genotype II, Serogroup 8. CVR profile analysis showed ASFV Genotype I with different variants, while Genotype II presented only one CVR variant. This is the first report of ASFV Genotype II in Burkina Faso, Cote d'Ivoire, and Mali. The introduction of ASFV Genotype II and its co-circulation with Genotype I in pig populations in these West African countries threatens food security and complicates control measures. Therefore, increased surveillance at international ports of entry, restrictions on live pig movements within the countries, and improved farm-level biosecurity measures are needed to control the further spread of the disease.

## 1. Introduction

African swine fever (ASF) is a highly lethal and incurable disease affecting domestic pigs and wild boars, caused by the ASF virus (ASFV) [1]. ASF has a significant economic impact on pork production, food security, and international trade in endemic areas [2]. Due to its transboundary nature, ASF spreads rapidly, leading to severe financial losses [3]. The first documented report of ASF was in 1921 in Kenya by a British Veterinary Pathologist following disease outbreaks among pigs [4]. Subsequently, the disease was reported in other African countries [5, 6]. In 1957, ASFV Genotype I was introduced to Europe via Portugal; however, it was the 1960 outbreak that spread to other European countries. Although it has been eradicated in the affected territories, including the recent ASF-free declaration on the Island of Sardinia in Italy, ASFV Genotype II was reintroduced into Europe via Georgia in 2007 [7–9]. Currently, ASF has been reported in over 20 European countries in either domestic and or wild pigs [10, 11]. In recent years, ASF has also spread to several countries in Asia, including the world's largest pork-producing nation, China, in 2018 [12–14].

The ASFV is a complex DNA virus, ranging in size from 170 to 190 kb, and is classified as the sole member of the family Asfarviridae and the genus *Asfivirus* [15]. Although 24 genotypes of ASFV had been described based on the partial characterization of the B464L gene [16–18], a recent study demonstrated Genotype XVIII as a mixture of Genotype I and VIII, thus, confirming only 23 ASFV genotypes [19]. Most of these ASFV genotypes are linked to regions where the sylvatic and tick transmission cycles have been identified, particularly in East and Southern Africa [6, 20, 21]. In West Africa, Genotype I was the only circulating ASFV genotype, until the recent report of Genotype II in Nigeria [22]. In Europe, the recent ASF outbreaks are caused by Genotype II [23, 24]. In Asia, both Genotypes I and II are circulating and are responsible for ASF outbreaks [12, 25]. Currently, Genotype II is likely the most widely distributed ASFV genotype, having been reported in Africa, Asia, Europe, and Oceania [26].

The ASFV Genotype I was first reported in West Africa in Senegal in 1959, followed by frequent outbreaks in the country from the late 1980s to the 2000s [27]. In 1982, it was reported in Cameroon [28]. During the epidemic in the 1990s, ASF was reported in several West African countries, including Guinea in 1992, Cabo Verde in 1993, Côte d'Ivoire in 1996, The Gambia in 1997, Benin in 1997, Nigeria in 1998, Burkina Faso in 2003, and later in Mali in 2016 [5, 29, 30]. The disease is endemic in sub-Saharan countries and has been reported in most pig-producing regions and production systems [31, 32]. While most reports of ASF have been in domestic pigs, the disease has also been reported in giant forest hogs, suspected to be a spillover case, while warthogs remain asymptomatically infected and play a key role in virus amplification [21, 33, 34]. Livestock movement and the trade of live animals and pig products between neighboring countries in West Africa is common and plays a key role in ASF outbreaks. For instance, the first ASF outbreak in Nigeria was traced to pigs trading along international borders with the Benin Republic [35]. This movement poses a high risk of introducing new diseases or strains/variants of etiological agents into new areas. Due to cultural practices, poor pig slaughter facilities, and high demand for live pigs, the marketing of pigs in sub-Saharan countries is via live animal markets [31, 36, 37]. In addition, weak veterinary and surveillance systems in most countries lead to easy and unrestricted movement of sick animals, thereby enhancing disease spread and complicating control measures [38].

Since 2017, ASF outbreaks have been reported in several West African countries, including Côte d'Ivoire (2017–2021), Burkina Faso (2019–2020), Nigeria (2019–2021), Ghana (2022), and Mali (2022–2023). In Nigeria, several states, particularly in the southern parts, experienced initial outbreaks of suspected ASF in backyard intensive, clustered, and commercial pig farms. These outbreaks were characterized by high morbidity and severe mortality rates. The ASF outbreak in Burkina Faso was first reported in the Meguet district of the Ganzourgou province. This study investigated the ASFV genotype responsible for the outbreaks in four West African countries—Nigeria, Burkina Faso, Côte d'Ivoire, and Mali—from 2017 to 2023 and its implication for pork production and food security.

## 2. Materials and Methods

### 2.1. Study Area and Sample Collection

Nigeria, Burkina Faso, Côte d'Ivoire, and Mali are all located in West Africa. Nigeria is divided into 36 states: 19 states in the north and 17 in the south, while Burkina Faso, Côte d'Ivoire, and Mali have 45, 31, and 19 provinces, respectively. In Nigeria, ASF samples were collected between 2019 and 2021. In 2019, ASF outbreaks were reported in plateau and cross river states. Starting from February 2020, ASF outbreaks were reported in a pig farming cluster in Oke-Aro and Lagos state, which persisted until December 2020 and spread to other states including Akwa Ibom, Abia, Anambra, Edo, Delta, Ogun, Oyo, Osun, and Kaduna States, due to the movement and sale of live pigs. Whole blood, sera, and tissue samples were collected from sick and dead pigs in affected farms and submitted to the National Veterinary Research Institute Vom, Nigeria, for analysis.

**Figure 1:**
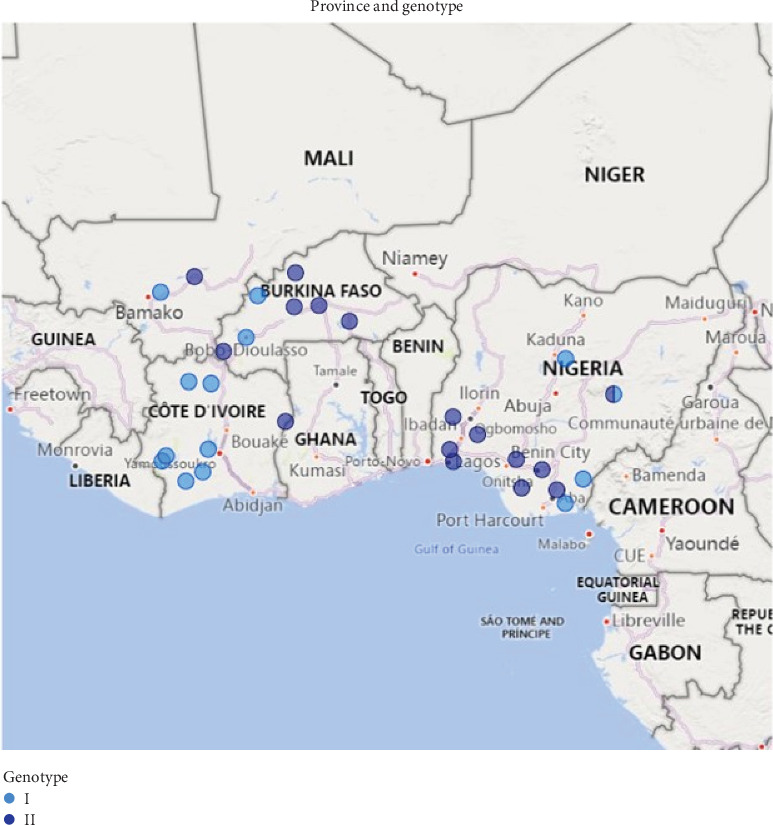
Map of West Africa highlighting the regions within the four countries with ASFV Genotype I (sky blue) and II (dark blue) outbreaks between 2017 and 2023.

Burkina Faso comprises 45 provinces. Initial suspected ASF cases were reported between July and December 2019 in farms located in the Kossi and Houet provinces. Between March and July 2020, more ASF cases were reported in five additional provinces. In Côte d'Ivoire, ASF outbreaks were reported between September 2017 and April 2021 in eight out of 31 provinces. In Mali, the ASF outbreaks were reported in two provinces between September 2022 and March 2023 (Figure 1). In Côte d'Ivoire, samples for analysis were collected from the spleen, kidney, liver, and lungs of pigs, while tissue and blood samples were collected from clinical cases in Mali and Burkina Faso.

### 2.2. Laboratory Diagnosis

Samples were processed and viral DNA was extracted using AllPrep DNA/RNA/Protein Mini Kit (Qiagen Hilden, Germany) following the manufacturer's protocol. ASF was diagnosed by detecting ASFV using real-time polymerase chain reaction (qPCR) as previously described [39].

### 2.3. Molecular Characterization of ASFV

Selected ASFV positive samples were characterized by targeting five genes: the partial the B646L gene encoding the p72 protein, full-length E183L gene encoding the p54 protein, central variable region (CVR) within B602L gene, intergenic region (IGR) between I73R and I329L genes, and the partial EP402R (CD2v) gene. The targets were amplified as previously described [40–43]. The PCR products were sequenced using the Sanger sequencing method. The raw sequences were assembled using the Vector NTI software (Invitrogen) version 11.5 and BioEdit (Hall, 1999) using default settings. Confirmation of sequence identities was carried out using the BLAST tool (https://blast.ncbi.nlm.nih.gov/Blast.cgi) and phylogenic trees were constructed on MEGA X using the neighbor-joining method for p72, minimum evolution method for p54, and maximum-likelihood method for CD2v [44]. ASFV reference sequences were retrieved from the GenBank and included in the construction of p72, p54, and CD2v phylogenetic trees. The multiple sequence alignments and analysis of the IGR were performed using BioEdit. To deduce the CVR amino acid tetramers and profiles, the nucleotide sequences were translated to protein sequences using an in-house python script on the Spyder IDE.

## 3. Results

### 3.1. Outbreak Investigations and Molecular Detection of ASFV

The ASF outbreaks in the four West African countries, Nigeria, Burkina Faso, Côte d'Ivoire, and Mali clinically affected about 36,087, 665, 7342, and 700 pigs, respectively, from different provinces/states (Table 1). The overall morbidity rate in the four countries was very high, ranging from 86% to 100%. However, the mortality rates varied in each country: Burkina Faso (13.3%–83.5%), Nigeria (1.6%–91.7%), Côte d'Ivoire (39.7%–100%), and Mali (50%–75%). From the total number of samples collected from each country, the ASFV genome was detected by qPCR in 151/226 (66.8%), 23/32 (71.8%), 29/35 (82.9%), and 23/23 (100%) in Nigeria, Burkina Faso, Côte d'Ivoire, and Mali, respectively. Further characterization of the ASFV was performed on representative positive samples with the lowest *C*q values from different regions in each country (Table 1).

### 3.2. Phylogenetic Analysis of the ASFV Isolates

**Figure 2:**
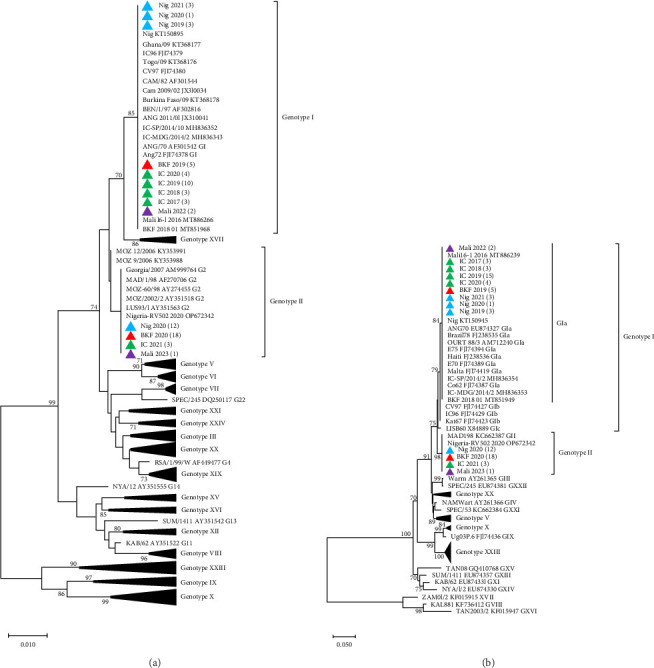
Phylogenetic tree of (A) partial *B646L* (*p72*) gene nucleotide sequences inferred using the neighbor-joining method and (B) the full-length p54 gene sequences based on the minimum evolution method, with the evolutionary distances computed using the Kimura two-parameter method. The ASFV isolates analyzed in this study from 2017 to 2023 are shown in red (Burkina Faso), green (Côte d'Ivoire), blue (Nigeria), and purple (Mali), with the number of sequences generated in various years from each country indicated in brackets. Only the bootstrap values greater than 70% are shown.

Phylogenetic analyses of p72 gene sequences, with representatives retrieved from GenBank, revealed that ASFV samples collected from Côte d'Ivoire (2017–2020), Nigeria (2019 and 2021), Burkina Faso (2019), and Mali (2022) clustered with ASFV Genotype I. These samples grouped with the previously reported ASFV strains circulating in Burkina Faso (KT368178), Nigeria (KT150878), Côte d'Ivoire (FJ174379), Mali (MT886266), and other neighboring countries such as Ghana, Benin, Togo, and Cameroon. Conversely, ASFV samples collected in Nigeria (2020), Burkina Faso (2020), Côte d'Ivoire (2021), and Mali (2023), clustered with Genotype II and with ASFV strains reported in Mozambique (AY351518), Madagascar (AF270706), Zambia (AY351563), and Georgia (AM999764; Figure 2A). Phylogenetic analysis of the ASFV p54 gene sequences confirmed the clustering of the newly sequenced isolates from the four West African countries (Figure 2B).

**Figure 3:**
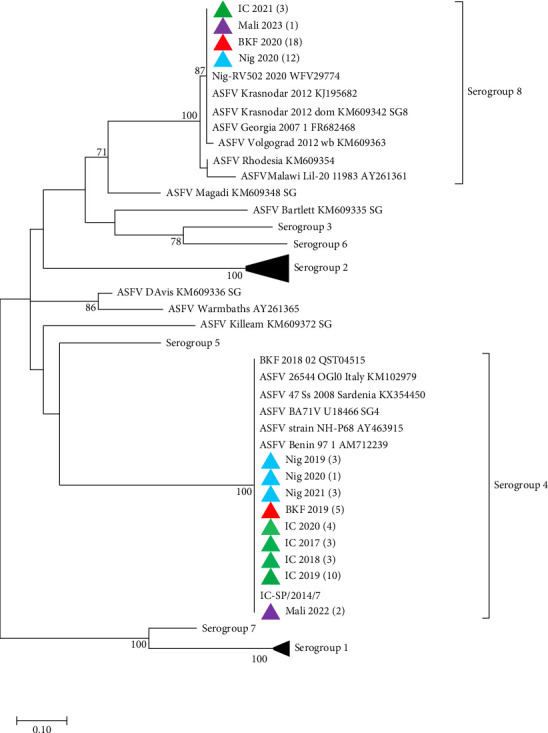
Maximum likelihood phylogenetic tree based on the partial amino acid sequence of the CD2v protein, showing the relationships between the ASFV isolates analyzed in this study, in red (Burkina Faso), green (Côte d'Ivoire), blue (Nigeria), and purple (Mali) and representatives of known ASFV serogroups as well as ASFVs clustering outside the eight established serogroups. The number of sequences generated from samples collected from various years from each country is indicated in brackets. The general reversible chloroplast model with gamma distribution was used. Only the bootstrap values greater than 70% are shown.

To determine the serogroups of the newly sequenced isolates from West Africa, phylogenetic analysis based on CD2v amino acid sequences showed that the isolates from Côte d'Ivoire, Nigeria, Burkina Faso, and Mali that clustered in ASFV Genotype I, belong to Serogroup 4, while those in Genotype II belong to Serogroup 8 (Figure 3). All the sequences from this study have been deposited in GenBank with accession numbers OQ164415–OQ164611 and PQ878212–PQ878319.

### 3.3. Sequence Analysis of CVR and the IGR Between I73R and I329L Genes

**Figure 4:**
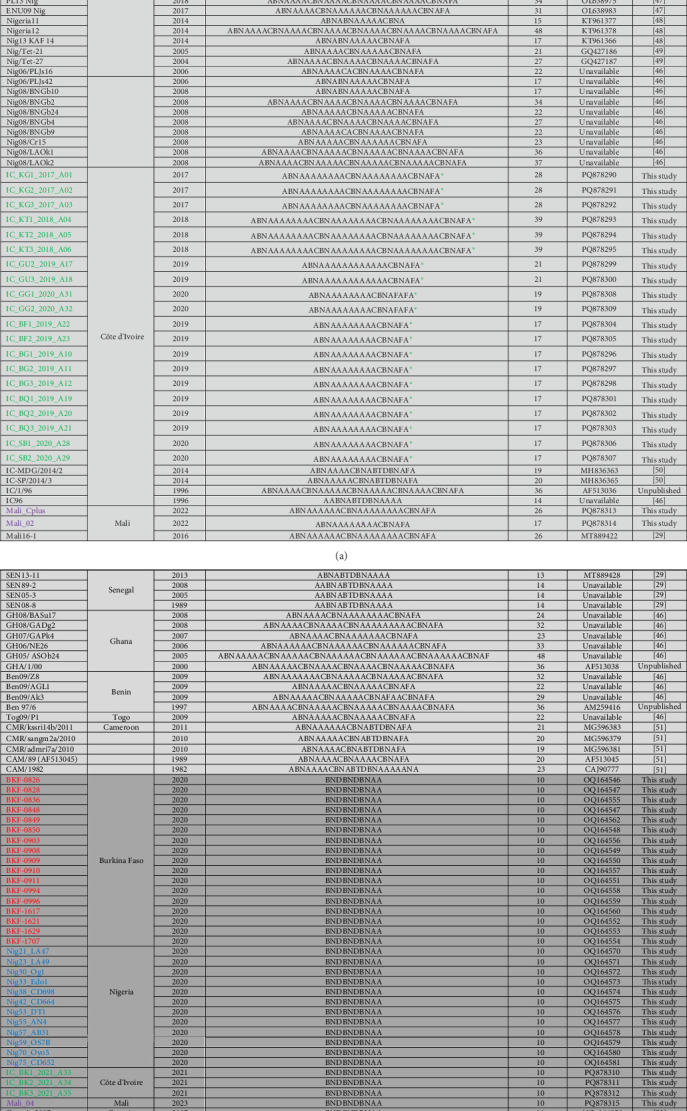
(A and B) Central variable region (CVR) profiles of ASFV Genotype I in light gray and Genotype II in dark gray from Burkina Faso (red), Nigeria (blue), Côte d'Ivoire (green), Mali (purple), and selected ASFV isolates from previous studies. The unique CVR profiles identified in this study are marked by asterisk (*⁣*^*∗*^). *Note*: CVR codes as previously described: CAST/CVST/CTST/CASI = A, CADT/CTDT/CADI/CAGT = B, GAST/GANT = C, CASM = D, CANT/CAAT = F, CTNT = G, NEDT = M, NVDT/NVGT/NVDI/NCDT = N, NANI/NADI/NASI = O, RAST = H, SAST = S, NVNT = T, NAST/NADT/NANT/NAVT = V, and SADT/SVDT = W.

**Figure 5:**
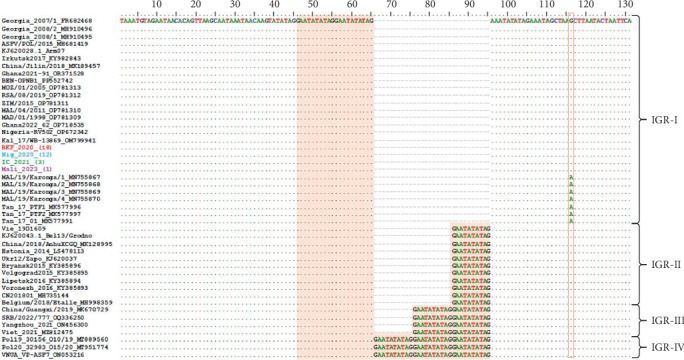
Multiple sequence alignments of the intergenic region (IGR) between I73R and I329L genes of the ASFV Genotype II isolates including those from Burkina Faso (red), Côte d'Ivoire (green), Nigeria (blue), and Mali (purple). The number of sequences generated from each country is indicated in brackets. The highlighted areas show the 10 bp tandem repeats and the nucleotide substitution between the isolates. The dots indicate the identical nucleotides in the alignment.

After translating the CVR sequences generated in this study into amino acids and coding them to the corresponding signature, the CVR tetrameric repeats of ASFV that caused the outbreaks in Nigeria, Burkina Faso, Côte d'Ivoire, and Mali between 2017 and 2023 were grouped based on previously described signature codes. All ASFV samples clustering in Genotype II/Serogroup 8 were homologous to those reported in Tanzania, China, Indonesia, and Mongolia. However, they presented a nonsynonymous T/C substitution compared to the reference strain Georgia 2007/1, as well as some isolates from Africa, Russia, and Asia, thereby maintaining an identical CVR profile (BNDBNDBNAA) within the tetrameric repeat region, except for the recently reported Genotype II isolates from Russian Far East (Figure 4 and Figure S1) [25, 29, 37, 43, 45–62]. The Genotype I/Serogroup 4 samples exhibited nine different CVR profiles, four of which were identical (including nucleotides and amino acids) to those previously reported in ASFV Genotype I in West Africa. All of the five new CVR profiles identified were found in Côte d'Ivoire, with one (ABNAAAAAAAACBNAFA) also present in Burkina Faso and Mali (Figure 4). Further analysis of the IGR between I73R and I329L genes classified the Genotype II ASFV samples, in IGR-I with two copies of the nucleotide repeat sequence (GAATATATAG), similar to Genotype II ASFV from Europe (Georgia; MH910496), Asia (China; MK189457), and Africa (Ghana, Madagascar, Mozambique, Zimbabwe, and Benin), except for those encountered in Tanzania and Malawi that have an A/G substitution (Figure 5).

## 4. Discussion

This study reports for the first time, the presence ASFV Genotype II in Burkina Faso, Cote Ivoire, and Mali. Additionally, ASFV Genotypes I and II were found to be co-circulating in Burkina Faso, Cote Ivoire, Nigeria, and Mali. Transboundary diseases like ASF are a constant threat to food security and livelihoods in endemic countries, including those in West Africa, resulting in substantial socioeconomic consequences [63]. ASF is the most lethal disease of pigs worldwide, with no cure or safe vaccine [64]. In West Africa, Nigeria has the largest pig population, with over 7 million pigs [65]. ASFV Genotype I was the only circulating genotype in West African countries until 2019. However, in 2020, ASFV Genotype II has emerged in the region, complicating the control measures against the disease. Investigations into these outbreaks revealed the introduction of Genotype II in West Africa: Nigeria (2020), Ghana (2022), and Benin (2023) [22, 57]. This current study reports the rapid spread of ASFV Genotype II from the index cases in Lagos State, Nigeria, to seven southern states of Nigeria within 10 months in 2020 and its first introduction into other West African countries: Burkina Faso (2020), Côte d'Ivoire (2021), and Mali (2023).

The initial outbreaks in Burkina Faso (2019), Nigeria (2019), Côte d'Ivoire (2017–2020), and Mali (2022) were confirmed to be caused by ASFV Genotype I through molecular characterization of the B646L (p72) and E183 (p54) genes, which was the only circulating ASFV genotype in Central and West Africa [5, 6]. Subsequently, Genotype II outbreaks occurred in Burkina Faso and Nigeria (2020), Côte d'Ivoire (2021), and Mali (2023). All Genotype II samples presented a similar CVR profile, whereas Genotype I samples exhibited several CVR variants. Moreover, the identification of a common CVR variant in ASFV Genotype I in Burkina Faso, Côte d'Ivoire, and Mali suggests an epidemiological link between the outbreaks in the these countries between 2019 and 2022, likely facilitated by their porous borders [66]. ASF is known to cause high morbidity and mortality rates, which can reach 100% in domestic pigs [67]. The outbreak in the four West African countries resulted in variable mortality rates, probably due to co-circulation of the low-virulent ASFV Genotype I that is endemic and the highly lethal Genotype II, which was introduced for the first time in the region [68]. The introduction route of ASFV Genotype II into West African countries remains unknown. However, given the porous nature of borders of Western countries its entry through the borders cannot be ruled out. For instance, in Nigeria, ASFV Genotype II was likely introduced via Lagos State, the most commercialized State in Nigeria, with portal entries by air and sea [22]. International travelers bringing in pork products or importing infected live pigs from other parts of Africa and Asia have been identified as routes through which ASFV was introduced into new European and Asian territories [10, 12, 69].

In Burkina Faso, the second largest pig farming nation in West Africa, the highest mortalities were recorded in the Cascade region, which has intense mining activities. Further investigations revealed that the ASFV outbreaks occurred after pigs were fed food waste from those mining sites, where foreigners with different food habits are present [70]. There was no evidence linking the 2020 ASF Genotype II outbreaks in different regions of Burkina Faso, as farms were owned by different owners. However, the outbreaks within the same areas were confined to a single location within the region, likely due to practices such as removing animals from infected farms, exchanging tools between breeders, gathering animals in live markets, or pig breeding practices that disregard biosafety rules [3, 71]. Although the ASFV Genotype II outbreak in Côte d'Ivoire was detected on a farm in the northeast (Bondoukou), near the Ghana border, there were no records of animal introduction or exchange with neighbors. However, this cannot be ruled out.

Further analysis of the IGR between I73R and I329L genes by sequence alignment of ASFV Genotype II circulating in Burkina Faso, Nigeria, Côte d'Ivoire, and Mali, showed that it is identical to Genotype II found previously in Europe (Georgia, Poland, and Armenia), Russia (Irkutsk and Kaliningrad), China (Jilin), Africa (Madagascar, Mozambique, Zimbabwe, and South Africa), and the recently reported Genotype II in Ghana and Benin [43, 56, 57, 72–75]. However, they differ from Genotype II isolates detected in Tanzania, Malawi, Russia (Bryansk, Volgograd, Lipetsk, and Voronezh), and European countries such as Estonia, Ukraine, and Belgium [43, 62, 76, 77].

The rapid spread of the ASF Genotype II in this study was likely facilitated by poor husbandry systems, particularly the clustering of farms, either built side by side or within a short distance of each other. Unpublished data from a 2019 serosurvey in some Nigerian states revealed antibodies against ASFV in pig farm clusters in Oke-Aro and Gerigbe, Lagos State. This finding suggests that the virus was circulating in pig farms, but was not reported by farmers. Therefore, it is likely that ASFV Genotype II might have been introduced into West Africa earlier than the confirmed 2020 index cases in Nigeria and Burkina Faso. The co-circulating of ASFV Genotype I and II in the four countries, where this study was carried out is very concerning, particularly with recent reports of highly lethal recombinant Genotypes I and II in China, Vietnam, and Russia [68, 78, 79]. The circulation of two ASFV genotypes and the possibility of a recombinant ASFV, in addition to the weak veterinary systems, complicates the control of the disease and threatens pig farming in West Africa. Therefore, whole genome sequencing and characterization of the recent ASFV genotypes circulating in the region could expand the knowledge on the genetic diversity and evolution dynamics of ASFV.

## 5. Conclusion

The study confirms for the first time, the presence ASFV Genotype II in Burkina Faso, Cote Ivoire, and Mali. In addition, our finding also confirms that ASFV Genotypes I and II were co-circulating in Burkina Faso, Cote Ivoire, Nigeria, and Mali between 2019 and 2023, resulting in severe mortalities and economic losses in these West African countries. These findings have a significant impact on food security and complicates ASF control measures in West Africa. To mitigate further spread of ASFV and the introduction of new genotypes into the subregion, it is crucial to increase surveillance at international ports of entry, restrict the movement of live pigs within these countries, and improve biosecurity measures at the farm level.

## Figures and Tables

**Table 1:** Characteristics of ASF outbreaks in Nigeria, Burkina Faso, Côte d'Ivoire, and Mali between 2017 and 2023, and the samples analyzed in this study.

Country	Province/state	Location	No. of susceptible cases	Morbidity rate (%)	Mortality rate	Date of collection	Sample type	RT-PCR (Cq)	Sample ID
Burkina Faso	Kossi	Bomborokuy	21	100	42.9	July 2019	Blood	18.47	BKF-19/0320/0352
	Houet	Bobo Dioulasso	33	100	18.2	October 2019		18.37	BKF-19/0431/0494
		Toussiana	9	100	44.2	December 2019		17.55	BKF-19/0906/3898
			14	100	50.0			17.17	BKF-19/0908/3903
								14.71	BKF-19/0908/3904
	Kadiogo	Songdin	30	100	13.3	March 2020		15.24	BKF-20/0185/0826
								15.59	BKF-20/0185/0828
			35	100	80.0	April 2020		14.67	BKF-20/0197/0910
								15.92	BKF-20/0198/0911
								24.14	BKF-20/0213/0994
								18.33	BKF-20/0213/0996
	Léraba	Fourkoura	162	95.0	80.2	April 2020		22.10	BKF-20/0186/0836
								17.04	BKF-20/0186/0848
								18.33	BKF-20/0186/0849
								19.80	BKF-20/0186/0850
	Boulgou	Tenkodogo	158	100	83.5	April 2020		27.33	BKF-20/0195/0903
			30	100	73.3			22.59	BKF-20/0196/0908
								17.19	BKF-20/0196/0909
	Yatenga	Namissiguima	37	86.0	81.1	May 2020		15.73	BKF-20/0339/1617
			56	100	17.9			17.39	BKF-20/0340/1621
			40	100	32.5	June 2020		14.92	BKF-20/0341/1628
								14.48	BKF-20/0341/1629
	Sanguié	Didyr	40	100	47.5	July 2020		15.45	BKF-20/0372/1707

Nigeria	Cross River state	Ikom	Abattoir sample	ND	ND	2019	Spleen	18.35	Nig2_CR65T
	Cross River state		Abattoir sample	ND	ND	2019	Spleen, liver	22.25	Nig3_CR58T
	Plateau state	Shenam	Abattoir sample	ND	ND	2019	Spleen, liver	16.77	Nig58_SH383
	Akwa Ibom state	Uyo	10	100	30.0	2020	Spleen	20.95	Nig37_AK5
	Lagos state	Oke Aro New site	29,811	ND	49.7	February 2020	Liver, spleen	23.30	Nig21_LA47
								18.63	Nig23_LA49
	Ogun state	Ewekoro	580	ND	57.8	August 2020	Blood, liver	21.55	Nig30_Og1
	Edo	Ikopba-Oklia	250	ND	1.60	August 2020	Blood	17.19	Nig33_Edo1
	Oyo state	Ogbomosho	300	100	32.7	2020	Liver	21.40	Nig38_CD698
	Plateau state	Jos	12	100	91.7	2020	Spleen	22.18	Nig42_CD664
	Plateau state		3	100	33.3	2020		26.39	Nig75_CD652
	Delta state	Asaba	750	ND	85.9	August 2020		26.78	Nig53_DT1
	Anambra	Aguata	370	ND	40.5	August 2020		19.74	Nig55_AN4
	Abia	Ubakala	3394	94.9	80.1	2020		19.57	Nig57_AB31
	Osun	Osogbo	79	ND	49.4	2020		17.99	Nig59_OS7B
	Oyo state	Apata	500	ND	10.0	2020		24.43	Nig70_Oyo5
	Kaduna state	Gwantu	19	100	42.1	2021		16.92	Nig64_GW2
	Kaduna state	Fadan Kagoma	9	100	55.6	2021		13.35	Nig65_217B
	Kaduna state	Kafanchan	Abattoir samples	ND	ND	2021		18.12	Nig67_218I

Côte d'Ivoire	Region du Poro	Korhogo	431	100	67.5	September 2017	Spleen, kidney, liver, lung	20.10	IC_KG1_2017
								20.23	IC_KG2_2017
								18.94	IC_KG3_2017
	Region de la Bagoué	Kouto	ND	ND	ND	October 2018		21.08	IC_KT1_2018
								22.15	IC_KT2_2018
								23.36	IC_KT3_2018
	Region du Guemon	Bangolo	1500	100	100	August 2019		16.32	IC_BG1_2019
			500	100	100			20.22	IC_BG2_2019
	Region de Cavally	Guiglo	213	100	95.8	October 2019		22.16	IC_GU1_2019
								20.70	IC_GU2_2019
								19.09	IC_GU3_2019
		Blolequin	ND	ND	ND	October 2019		21.49	IC_BQ1_2019
								18.42	IC_BQ2_2019
								23.72	IC_BQ3_2019
	Region de la Marahoué	Bouaflé	ND	ND	ND	October 2019		26.10	IC_BF1_2019
								19.07	IC_BF2_2019
								21.66	IC_BF3_2019
	Region du Guemon	Douekoué	4564	100	76.6	October 2019		18.93	IC_NK1_2019
								17.71	IC_NK2_2019
								23.14	IC_NK3_2019
	Region du Gôh	Gagnoa	ND	100	100	February 2020		19.29	IC_GG1_2020
								22.17	IC_GG2_2020
	Region de Nawa	Soubré	ND	100	100	February 2020		20.75	IC_SB1_2020
								22.05	IC_SB2_2020
								21.10	IC_SB3_2020
	Region de Gontougo	Bondoukou	71	100	70.4	April 2021		20.71	IC_BK1_2021
			63	100	39.7			18.58	IC_BK2_2021
								22.54	IC_BK3_2021

Mali	Koulikoro	N′Tionon	200	100	50.0	September 2022	Tissue	25.81	Mali_537/C+
			200	100	75.0			25.51	Mali_545/C02
	San	Bountenisso	300	100	66.7	March 2023		21.39	Mali_308/C04

Abbreviation: ND, not determined.

## Data Availability

DNA sequences generated and analyzed in the current study are available in GenBank under accession numbers OQ164415–OQ164611 and PQ878212–PQ878319.

## Supporting Information

Supporting Information Figure S1: Multiple sequence alignments of (A) nucleotides and (B) amino acids of the central variable region (CVR) within B602L of the ASFV Genotype II isolates, including those from Burkina Faso (red), Côte d'Ivoire (green), Nigeria (blue), and Mali (purple). The number of sequences generated from each country is indicated in brackets. The nucleotide/amino acid substitution between the isolates are in blocks, while the tetrameric repeat region is highlighted in gray.
