# Supplementary material for: Detection of African Swine Fever Virus Genotype II in West Africa (2020) and Its Co-Circulation With Endemic Genotype I: Implications for Pig Production
Source: Transbound Emerg Dis. 2025 Jun 10;2025:5396227. doi: 10.1155/tbed/5396227 (PMC12173545; doi:10.1155/tbed/5396227)

Supplementary Figure S1: Multiple sequence alignments of (A) nucleotides and (B) amino acids of the central variable region (CVR) within B602L of the ASFV genotype II isolates, including those from Burkina Faso (red), Côte d'Ivoire (green), Nigeria (blue), and Mali (purple). The number of sequences generated from each country is indicated in brackets. The nucleotide/amino acid substitution between the isolates are in blocks while the tetrameric repeat region is highlighted in grey.

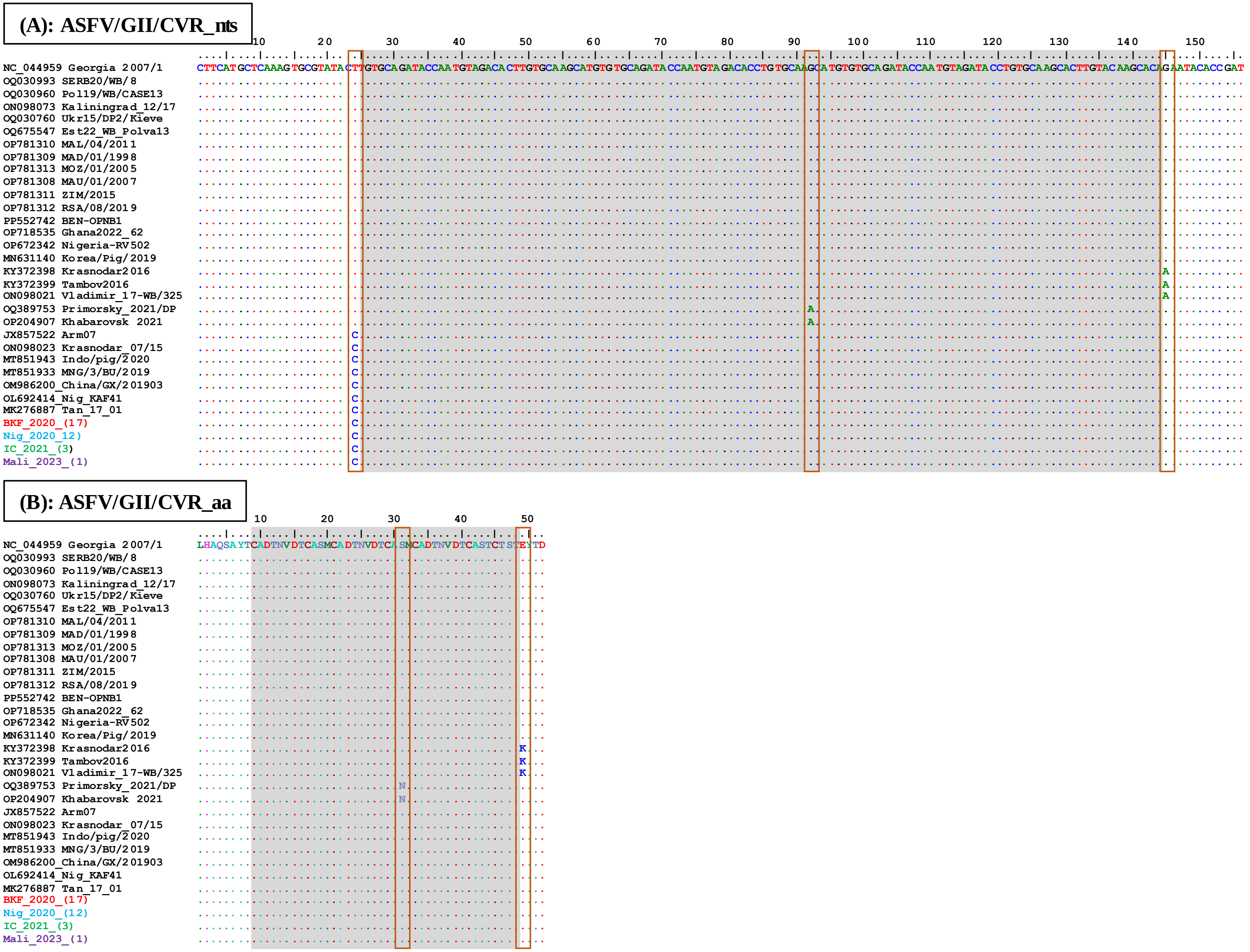

Supplement: Supporting Information — Figure S1: Multiple sequence alignments of (A) nucleotides and (B) amino acids of the central variable region (CVR) within B602L of the ASFV Genotype II isolates, including those from Burkina Faso (red), Côte d'Ivoire (green), Nigeria (blue), and Mali (purple). The number of sequences generated from each country is indicated in brackets. The nucleotide/amino acid substitution between the isolates are in blocks, while the tetrameric repeat region is highlighted in gray. [file 5396227.f1.pdf]
